# Fault Types and Diagnostic Methods of Manipulator Robots: A Review

**DOI:** 10.3390/s25061716

**Published:** 2025-03-10

**Authors:** Yuepeng Zhang, Jun Wu, Bo Gao, Linzhong Xia, Chen Lu, Hui Wang, Guangzhong Cao

**Affiliations:** 1 School of Sino-German Robotics, Shenzhen Institute of Information Technology, Shenzhen 518172, China; ypzhang@sziit.edu.cn (Y.Z.); gaob@sziit.edu.cn (B.G.); xialz@sziit.edu.cn (L.X.); 2 Inovance Industrial Robot Reliability Technology Research Institute, Shenzhen Institute of Information Technology, Shenzhen 518172, China; luchen@sziit.edu.cn (C.L.); wanghui@sziit.edu.cn (H.W.); 3 Guangdong Key Laboratory of Electromagnetic Control and Intelligent Robots, Shenzhen University, Shenzhen 518060, China; gzcao@szu.edu.cn

**Keywords:** manipulator robot, type of fault, signal acquisition, fault diagnosis

## Abstract

Manipulator robots hold significant importance for the development of intelligent manufacturing and industrial transformation. Manufacturers and users are increasingly focusing on fault diagnosis for manipulator robots. The voltage, current, speed, torque, and vibration signals of manipulator robots are often used to explore the fault characteristics from a frequency perspective, and temperature and sound are also used to represent the fault information of manipulator robots from different perspectives. Technically, manipulator robot fault diagnosis involving human intervention is gradually being replaced by new technologies, such as expert experience, artificial intelligence, and digital twin methods. Previous reviews have tended to focus on a single type of fault, such as analysis of reducers or joint bearings, which has led to a lack of comprehensive summary of various methods for manipulator robot fault diagnosis. Considering the needs of future research, a review of different fault types and diagnostic methods of manipulator robots provides readers with a clearer reading experience and reveals potential challenges and opportunities. Such a review helps new researchers entering the field avoid duplicating past work and provides a comprehensive overview, guiding and encouraging readers to commit to enhancing the effectiveness and practicality of manipulator robot fault diagnosis technologies.

## 1. Introduction

With the rapid development of intelligent manufacturing, manipulator robots are gradually replacing traditional manual operations, significantly improving production efficiency while reducing labor costs [1,2,3,4]. They have become one of the key technologies in the intelligent manufacturing industry due to their high efficiency, high precision, and high stability. However, in automated production, robots at each stage have a clear division of labor and close collaborative relationships [5,6,7]. When a robot malfunctions, it will inevitably affect the progress and safety of production [8]. The field of manipulator robotics is experiencing a notable increase in demand for research into fault diagnosis [9]. Firstly, there is a demand for increased production efficiency. Manipulator robots, as key production tools in modern manufacturing, directly impact production efficiency and product quality due to their operational status [8,10]. Through fault diagnosis in manipulator robots, potential issues can be detected promptly, thereby preventing sudden equipment failures that lead to production downtime and consequently enhancing production efficiency [11]. Secondly, there is a need to reduce maintenance costs. Fault diagnosis of manipulator robots enables the extension of maintenance and overhaul intervals, thereby reducing the operation and maintenance costs for enterprises [12,13]. Thirdly, there is a demand for the intelligent upgrading of production. Fault diagnosis technology for manipulator robots is a key element in this process. It can enhance the technological autonomy and controllability, as well as the level of intelligence of manipulator robots, thereby satisfying the ever-increasing production demands of enterprises [14]. Fourthly, there is a demand for safety assurance. The operational environments of manipulator robots are typically complex and challenging. Through fault diagnosis, monitoring the operational status and providing early warnings for abnormal conditions can prevent the occurrence of safety accidents [15].

The application of advanced fault diagnosis techniques to manipulator robot systems not only improves the operational efficiency and performance of the system [16,17], but also enhances its reliability by optimizing the fault diagnosis process [18,19]. Fault diagnosis methods for manipulator robots include data mining, machine learning, neural networks, and digital twins, among others, and have yielded some research outcomes [20,21,22,23,24,25]. For instance, Wei Bo et al. [24] conducted an oil sample analysis by extracting the mixed lubricating oil from the Rotary Vector (RV) reducer to investigate the relationship between rotational speed and load with the properties of the lubricating oil, thereby determining the operational status of the robot. Yan Bei et al. [25] employed a combination of horizontal-level functions and gas volume techniques to estimate multiple performance indicators of manipulator robot lubricating oil.

Some robot companies have conducted long-term research and practice in the field of fault diagnosis [26]. For example, well-known robot manufacturers such as FANUC, KUKA, ABB, etc. have leading technologies and solutions in fault diagnosis of manipulator robots [27,28]. In addition, some universities and research institutions have also explored the fault diagnosis of manipulator robots [29,30,31,32,33,34]. Ref. [32] focuses on the potential actuator faults in the joints of heavy-duty manipulator robots during operation. It investigates a data-driven intelligent fault diagnosis algorithm, which involves injecting various faults into the established dynamics model of the heavy-load manipulator robot to obtain unbalanced samples. Ultimately, a composite neural network is employed to achieve the diagnosis and identification of faults. Ref. [33] investigates fault detection methods for manipulator robot gearboxes based on vibration signals. This method employs the time-domain averaging of phase to remove estimated deterministic signals from the measured vibration signals, thereby calculating the residual signal and achieving fault diagnosis. Ref. [34] investigates a fault diagnosis method for manipulator robot joint bearings based on acoustic signal analysis. This method extracts defect frequency peaks and changes in bearing rotational speed from the original acoustic signals using the envelope spectrum and then employs a convolutional neural network (CNN) to extract features and classify the defects.

The fault diagnosis of manipulator robots is meaningful for improving the development of industrial automation. For fault diagnosis in manipulator robots, there are some questions worth considering: What are the faults of existing manipulator robots? What are the methods and techniques for fault diagnosis? How can existing technologies be combined to solve data quality, generalization ability, and interpretability issues, to improve the accuracy and practicality of fault diagnosis? What are the advantages and limitations of existing fault diagnosis methods? What are the future expectations of manipulator robot fault diagnosis? This paper provides a systematic review of the types of failures in manipulator robots, as well as the methods and techniques used for fault diagnosis. Compared to other reviews, this review has two differences:

(1) Previous reviews have not classified the types of faults and the development of diagnostic methods, but instead mostly analyzed the faults of a certain component of manipulator robots. For example, Huang et al. [35] discussed the diagnosis of compound faults in rotating machinery, and Nandi et al. [36] reviewed the state monitoring and fault diagnosis of motors, but did not analyze diagnostic methods and techniques. This review provides general guidance on this topic.
(2) Previous reviews have not discussed and analyzed the overall structure of manipulator robots and the types of failures that can occur in each component. This review supplements this content.

As a comprehensive review dedicated to summarizing and discussing the types of faults and fault diagnosis methods in manipulator robots, this paper has made the following contributions: (1) This paper analyzes the composition of manipulator robot systems and the types of faults in various components, including Rotary Vector (RV) reducers, balance cylinders, motors, and joint bearings. (2) This paper systematically summarizes the methods and techniques involved in fault diagnosis of manipulator robots, categorizing them into traditional and modern approaches based on their development history. Furthermore, it provides a detailed classification and discussion of these methods based on their characteristics. (3) This paper macroscopically explored the advantages, challenges, and development trends of fault diagnosis technology for manipulator robots.

## 2. Composition and Fault Types of Manipulator Robots

### 2.1. Faulty Components and Fault Types in Manipulator Robots

**Figure 1:**
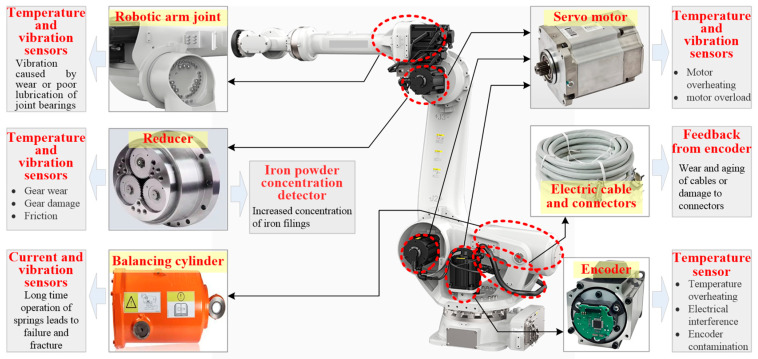
Components and fault characteristics of manipulator robots.

The precise structure of the core components of manipulator robots allows them to maintain stable performance under various working conditions and to handle industrial tasks that require high precision and high intensity. Once a critical component of a manipulator robot fails, it not only reduces the service life but also accelerates the degradation cycle of the robot. The basic components of a manipulator robot system involve reducers, counterbalance cylinders (only for heavy-duty manipulator robots), servo motors, robotic arms, encoders, control systems, cables and connectors, and lubrication systems, as shown in Figure 1. Among these components, servo motors, RV reducers, and joint bearings are the most common and susceptible to failure in manipulator robots. When the failure occurs, it directly impacts production efficiency and progress.

### 2.2. Fault Types of Manipulator Robots

The development process of fault diagnosis for manipulator robots has been of concern since the invention and manufacture of robots [37,38]. Initially, robot manufacturers and users relied on simple protections, such as inspections conducted by experienced engineers or regular production line shutdowns [39,40]. As the tasks performed by manipulator robots become increasingly complex, it has become very important to diagnose faults before they occur, as robot downtime outside of project execution plans can disrupt the overall project schedule and cause serious economic losses. In fault diagnosis of manipulator robots, there are indeed complex interactions between different fault types, which have a significant impact on the operating status and diagnostic strategies of the robot. The coupling fault between mechanical components and electrical systems leads to abnormal vibration of the entire system, which increases the complexity of fault diagnosis. The operating conditions of manipulator robots can affect the manifestation of faults, which puts higher demands on fault diagnosis models. Manipulator robot failures often do not occur in isolation, but gradually trigger chain failures of other components over time. For manipulator robot systems, the main causes of component failures are as follows, and summarized in Table 1.

**Reducer:** In the long-term operational state of the manipulator robot, the reducer in the joints may experience gear wear or damage due to frequent high/low-speed transitions, high torque loads, and prolonged transmission [41,42,43,44,45,46]. Rohan et al. [43] proposed a method for detecting and diagnosing mechanical faults in RV reducers using current signals from embedded control units. This method involves decomposing the original signal into wavelets and extracting, selecting, and injecting various types of features from them to enhance the accuracy of machine learning-based classifiers. Yang et al. [44] proposed a composite fault diagnosis method for harmonic reducers based on a deep capsule graph convolutional network (GCN). The method acquires fault signal spectra from multi-sensor data and constructs a relationship graph using an adjacency matrix. It then utilizes both a deep capsule network and a GCN to learn fault vector representations and the relationships between different single-label faults. Yang [45] proposed a method based on generative adversarial networks to address the issue of data imbalance. This method involves collecting vibration data and using a multi-scale CNN to achieve fault diagnosis of harmonic reducers. Xing et al. [46] proposed a one-dimensional CNN adaptive method based on matrix kernels, aiming at the characteristics of sampling signals in manipulator robots. This method adjusts the size of the convolutional kernels to process the data of multichannel fusion and to extract contextual features from continuous time-domain data.

**Joint bearing:** Poor lubrication of the joint bearings, rotor asymmetry, defects in the bearing seat rings, and defects of rolling elements can lead to unsmooth motion or vibration in manipulator robots [47,48,49,50,51]. Yazici et al. [47] reported an adaptive statistical time-frequency method for detecting bearing faults. This method is tested on defective bearings with scratches on the outer race, balls, and cages, and all defects are correctly classified. Xia et al. [48] proposed a deep perception adversarial domain adaptive method for fault diagnosis of manipulator robot bearings under variable operating conditions, which is superior to CNN and conditional domain adversarial networks. McInerny et al. [49] diagnosed bearing faults using envelope detection technology and demonstrated that this method is superior to traditional spectral analysis methods. Raouf et al. [50] used a feature aggregation network with a hierarchical information aggregation mechanism for fault detection of manipulator robot bearings and demonstrated its adaptability and robustness in different experimental scenarios. Zhang et al. [51] considered the rolling elements and bearing seats of bearings, established a dynamic model of rolling bearings compound fault based on time-varying displacement, and discussed the relationship between the vibration response of compound faults and three different operating conditions.

**Servo motor:** Servo motors are the power source for manipulator robots. Due to prolonged high-load operation, it may experience a fault due to overheating, overload, or damage to the motor encoder [52,53]. Junior et al. [52] measured acceleration data in two different directions, and used one-dimensional CNN to detect and diagnose six different types of motor faults. Suawa et al. [53] collected multi-source data such as motor vibration and sound, and analyzed and solved the problem of predictive maintenance of motors through artificial intelligence methods.

**Electric cable:** The abrasion and aging of cables will lead to electrical connection failure, affecting the normal work of manipulator robots [54,55]. Aiming at the damage of conductor or insulator in the cable due to different conditions, which leads to faults, Kim et al. [54] studied a new method for soft faults diagnosis of control cable based on detection, using the three-phase current to calculate the sum and ratio of current, and taking it as the input of diagnosis network to detect the cause of soft faults. Kim [55] calculated the three-phase current through the automatic encoder, calculated the difference fraction to detect the cable fault, and used the fault method combining long short-term memory (LSTM) and attention mechanism to estimate the fault severity through time-related information.

**Screw:** Due to the long time and many times of carrying objects, the wear of robot screw leads to the deterioration of health. Yang et al. [56] used the motor current signal to diagnose the health state of the ball screw of the manipulator robot, the Fisher score for feature selection, and logical regression and k-nearest neighbor for fault diagnosis.

**Compound fault:** The manipulator robot system contains many components, which are spatiotemporally related and need to cooperate to complete the work. Due to the poor working environment, improper operation, long-term work, and other reasons, the functional components of manipulator robots are prone to damage, resulting in compound faults in the robot system [35,57,58,59]. For example, He et al. [58] proposed an adaptive redundant multi-wavelet compound fault diagnosis method to address the complexity and diversity of compound faults and combined it with Hilbert transform demodulation analysis to effectively detect compound faults in gearboxes. Zhou et al. [59] proposed a compound fault diagnosis algorithm for manipulator robots based on multimodal feature extraction and fusion. This method uses a multi-head self-attention enhanced CNN module and an LSTM module to simultaneously learn fault-related features from different perspectives, fuse the extracted features, and perform compound fault classification.

**Table 1:** Fault locations, data types, and diagnostic methods for manipulator robots in common.

Position of Faults	Types of Data	Methods for Fault Diagnosis	References
Reducer	Current signal data	Based on wavelet features and statistical analysis	[43]
	Vibration signal data	Deep capsule graph convolutional network	[44]
	Vibration signal data	Generative adversarial networks and multi-scale convolutional neural networks	[45]
	Vibration signal data	Adaptive method of one-dimensional convolutional neural network	[46]
Joint bearing	Current data	Adaptive statistical time-frequency method	[47]
	Acceleration data	Deep perception adversarial domain adaptive method	[48]
	Vibration signal data	Envelope detection technology	[49]
	Current signal data	Feature aggregation network based on hierarchical information aggregation mechanism	[50]
	Vibration signal data	Four-degree-of-freedom dynamic model of compound local fault rolling bearings based on time-varying displacement	[51]
Servo motor	Vibration signal data (IMI uniaxial accelerometer)	One-dimensional convolutional neural network	[52]
	Vibration signal data, sound data	Deep convolutional neural networks, long-short term memory methods, and convolutional neural networks–long short-term memory	[53]
Electric cable	Current signal data	Calculate the total sum and ratio of three-phase currents	[54]
	Current signal data	Calculate the calculation difference fraction of the three-phase current	[55]
Screw	Current signal data	Fisher score, logistic regression, and k-nearest neighbor method	[56]
Compound fault	Acceleration data	Combining adaptive redundant multi-wavelet packet with Hilbert transform demodulation analysis	[58]
	Current signal data	Multi-head self-attention enhanced convolutional neural network module and long short-term memory network	[59]

In addition, sensors used in manipulator robots, such as encoders and force sensors, may also experience faults due to temperature changes, mechanical shocks, or electrical interference. For heavy-duty manipulator robots, the spring inside the balancing cylinder may also break due to prolonged operation. Table 1 summarizes the component positions, fault diagnosis data types, and diagnostic methods of common faults in manipulator robots.

However, in the actual working environment of manipulator robots, compound faults are the most common and strongly correlated with individual faults, rather than simply linear superposition. In addition, compound faults in manipulator robots are caused by the coupling of multiple subsystems and various types of noise, which makes it difficult to generalize them into common patterns, thus rendering their fault diagnosis more challenging [60,61].

### 2.3. Types of Fault Signal Acquisition

The common signals for collecting fault data of manipulator robots are mainly divided into two forms based on their collection paradigm and signal source. One type is the data generated by the robot itself during its operation, such as the current, voltage, output torque, angle, angular velocity of the robot joint motor, and the concentration of iron filings of the reducer wear [62]. Another type is to deploy sensors externally and collect available signals according to demand, such as robot vibration signals collected through high-precision accelerometers (vibration sensors), motor and reducer oil temperature collected through temperature sensors, and joint noise signals [63,64,65,66,67]. Among them, vibration signals are commonly used in the fault analysis of manipulator robots [68,69]. For instance, Islam et al. [70] collected data from manipulator robots using vibration sensors, preprocessed the vibration data, and then employed the gradient tree-based XGBoost machine learning classification algorithm to achieve fault diagnosis of manipulator robots. There is a distinct physical relationship between the faults of manipulator robots and vibration signals. When a robot faults, the vibrations generate additional torque components that affect the current spectrum, resulting in specific features in the frequency domain, which can be represented as [71]:

(1) $$f_{bec}=\left|f_{s}\pm kf_{r}\right|$$

where $f_{r}$ is the rotational frequency of the analyzed axis.

The faults can be modeled as additional torque components with amplitudes greater than the load at mechanical characteristic frequencies. This component is an additional component of the load torque $T_{L}$. The instantaneous torque applied to the motor is expressed as:

(2) $$T_{load}\left(t\right)=T_{L}+\Delta T_{load}\left(t\right)$$

where $T_{load}$ is the load torque.

It is worth considering that although vibration signals contain abundant information about the operational status of robots, the need for external sensors to measure vibrations and the requirement for higher-performance sensors due to frequency effects significantly increase costs. In addition, each system of manipulator robots contains multiple rigid and flexible components, and each joint requires multiple measurements, resulting in inaccurate diagnostic results and tedious work [72]. Some manipulator robots cannot use or install external sensors in actual industrial production environments, making it difficult to use vibration signals for manipulator robot fault analysis. Similarly, only collecting data from manipulator robots, such as voltage, current, speed, etc., cannot fully reflect fault information [73,74].

## 3. Fault Diagnosis Methods for Manipulator Robots

Fault diagnosis technology, as an important component of predictive maintenance, has become a crucial tool for ensuring the efficient, stable, and safe operation of robot systems in automated production. The fault diagnosis of manipulator robots is of great significance for the transformation and upgrading of the modern manufacturing industry.

Manipulator robot systems exhibit a wide variety of faults, and relying on conventional signal detection methods and fault diagnosis techniques has significant limitations. Faults in different components and locations cause varying changes, and their external performances are also diverse, representing a complex nonlinear mapping relationship. With the rapid development of data analysis technologies such as artificial intelligence and deep learning, obtaining high-precision robot fault signals through various sensors and measurement schemes is a basic prerequisite for fault diagnosis. The increase in methods for collecting various data information has led to a surge of heterogeneous information, there is a phenomenon of a large influx of heterogeneous information, characterized by overlap, contradiction, multi-source, and a wide variety of structures. There are significant differences in precision, detail, structure, and extraction methods, and these uncertain factors pose challenges to manipulator robot fault diagnosis.

There are various methods for diagnosing faults in manipulator robots, which can be divided into several stages based on the development process of diagnostic technology:

(1) The first method involves comparing the operational status parameters of manipulator robots, such as abnormal temperatures, vibrations, and noises, with their historical normal operating parameters to make maintenance decisions. This diagnostic method is simple and convenient, but accuracy is relatively low.
(2) The second method is to use the manipulator robot’s own state recognition and fault diagnosis mode to reflect fault information based on incorrect codes, making it easier for engineers to directly locate the type and location of the fault.
(3) The third method is to construct digital twins of virtual, utilizing actual manipulator robots and mapped proportional digital models, and implementing remote monitoring, providing fault diagnosis services, and maintenance support through the network.

**Figure 2:**
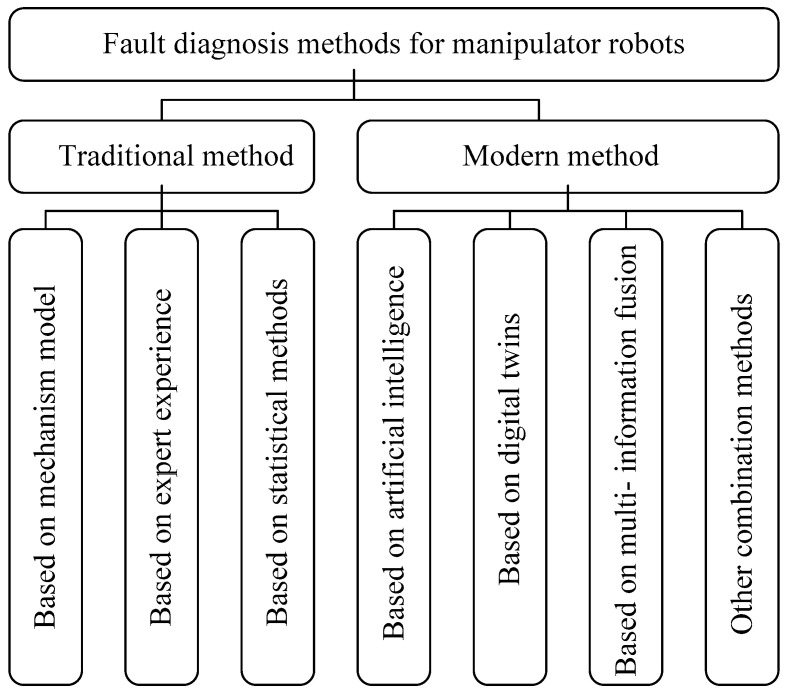
Classification of fault diagnosis methods for manipulator robots.

In addition, the research on fault diagnosis of manipulator robots can be classified according to their working principles and application technologies, and the application technologies can be further divided into traditional methods and modern methods, as shown in Figure 2. The traditional method usually utilizes expert experience, statistics, and other methods to use the knowledge and experience of early technical engineers to determine the cause of manipulator robot failures. Modern methods use new methods and technologies, such as machine learning, deep learning, and other artificial intelligence methods, as well as information fusion and digital twin to diagnose robot faults.

### 3.1. Based on Traditional Methods

#### 3.1.1. Method Based on Expert Experience System

**Figure 3:**
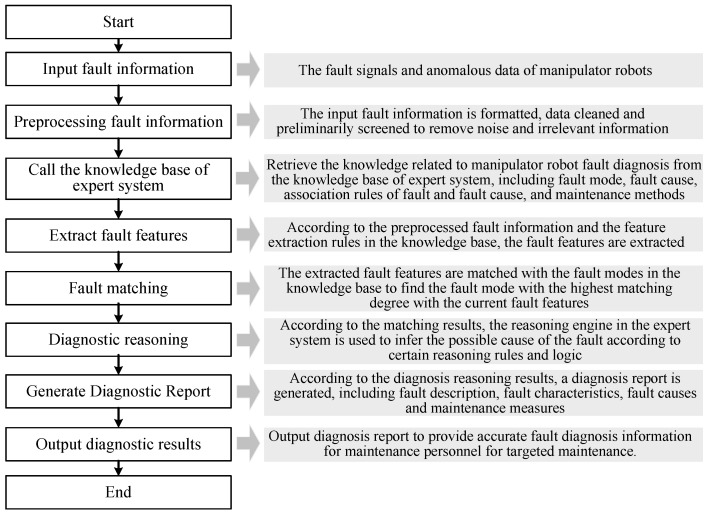
Fault diagnosis process of a manipulator robot based on an expert experience system.

The expert experience method is an early used method for manipulator robot fault diagnosis [31,75,76,77]. Its core is to use the experience and knowledge of experts with rich experience and professional knowledge to achieve fault diagnosis of manipulator robots, as shown in Figure 3. Liu et al. [76] first estimated the motion state of the robot using a Kalman filter and calculated the probability of each motion state corresponding to different fault modes, providing preliminary diagnostic results. They then selected appropriate knowledge from the expert system’s knowledge base to infer and judge the diagnostic results. Sun et al. [77] proposed a novel fault diagnosis method based on experimental data-driven stochastic fuzzy evidence acquisition and intuitionistic fuzzy set fusion. This method involves constructing a fuzzy expert system to match the samples under test. The intuitionistic fuzzy set fusion employs binary vector arrays of intuitionistic fuzzy data sets to represent the likelihood values of samples, capturing uncertainty information to enhance the reliability and accuracy of fault diagnosis.

Expert experience systems, while not requiring the establishment of precise mathematical models, heavily rely on expert empirical knowledge.

#### 3.1.2. Method Based on Statistical Models

By employing operations such as dimensionality reduction during data processing, redundant data can be effectively eliminated, significantly reducing the computational load. For instance, using multivariate statistical analysis methods, fault diagnosis can be performed based on the correlations between variables. Wu et al. [78] collected vibration signals from manipulator robots under three operating conditions as raw data. They employed principal component analysis, locally preserving projection, and isometric feature mapping to extract three-dimensional features from the vibration signals and used these features as the input for the training model, effectively identifying the fault types of manipulator robots.

Statistical methods are used in manipulator robot fault diagnosis to analyze historical data, identify features related to faults, and use these features to diagnose the faults that occur. For example, statistical indicators such as mean, median, variance, and residuals can be used to describe the operational data of manipulator robots.

#### 3.1.3. Method Based on Mechanism Knowledge-Driven Model

**Figure 4:**
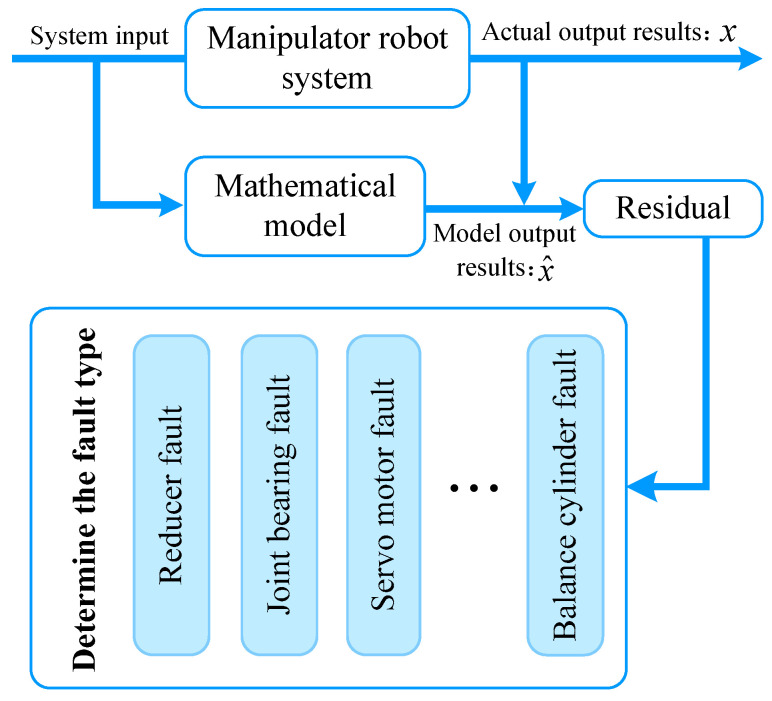
Framework of fault diagnosis method based on mechanism knowledge-driven model.

The fault diagnosis method based on a mechanism knowledge-driven model mainly relies on the physical model and accurate mathematical model of manipulator robots. It uses state estimation, and parameter estimation to diagnose faults by comparing and analyzing the difference information between the model output and the actual output [79], as shown in Figure 4.

For example, Piltan et al. [80] used a neural adaptive high-order variable structure observer to accurately estimate the position and torque signals of a manipulator robot and then utilized the characteristics of the position and torque signals of the manipulator robot to improve the robot’s fault recognition ability by support vector machines. They designed a modern fuzzy backstepping variable structure controller, used adaptive algorithms to fine-tune controller parameters, and reduced the impact of faults on manipulator robots. Moshgani et al. [81] used a method that combines an unknown input observer with a genetic algorithm to determine the optimal observer parameters and designed an adaptive threshold for the residual signal to achieve fault diagnosis of manipulator robots.

The Kalman filter is used for fault diagnosis of robots [82], which determines the possible existence of faults by comparing the residual between sensor measurements and Kalman filter predictions [83], as expressed in Equations (3)–(7).

- Prediction

Prediction of the state ahead, and prediction the error covariance

(3) x(k+1|k)=Ax(k|k)+Bu(k)

(4) $$p\left(k+1|k\right)=Ap\left(k|k\right)A^{T}+Q_{k-1}$$

- Correction

Compute the Kalman Gain

(5) $$K_{k}=\frac{H^{T}p\left(k+1|k\right)}{Hp\left(k+1|k\right)H^{T}+R\left(k\right)}$$

Update the estimated value, and update the prediction covariance

(6) $$x\left(k|k\right)=x\left(k|k-1\right)+K_{k}\left[z\left(k\right)-Hx\left(k|k-1\right)\right]$$

(7) $$p\left(k\right)=\left(I-K_{k}H\right)p\left(k-1\right)$$

where x(k|k) is the estimated value of time *k*, x(k|k−1) is the state estimation of time *k* − 1, x(k+1|k) is the predicted value of the next step, p(k) is the covariance matrix corresponding to the state variable, p(k+1|k) is the covariance matrix of the next step of prediction, *A* is the state transition matrix, *B* is the matrix, *H* is the observation matrix, *u* is the control input, and *Q* and *R* are the covariances.

For example, Van et al. [84] proposed a fault diagnosis method based on the Kalman filter to effectively detect actuator faults. This method calculates the estimation error of the Kalman filter (the difference between the actual output based on image features and the estimated output of the Kalman filter) and compares it with a preset threshold to determine whether the system has malfunctioned. Zhang et al. [85] proposed an efficient fault diagnosis and fault-tolerant control method based on adaptive extended Kalman filter and sliding mode control. This method identifies faults based on the control effectiveness coefficient estimated by the adaptive extended Kalman filter and verifies its fault diagnosis and control performance under various fault conditions through simulation.

Mechanism knowledge-driven fault diagnosis methods for manipulator robots focus on the system entity of the diagnosis object, with a solid theoretical foundation, but they demand high accuracy of the mechanism model.

#### 3.1.4. Methods Based on Signal Processing

Signal-based analysis and processing methods involve preprocessing data samples and then extracting time-domain, frequency-domain, and time–frequency-domain fault features from the data samples [86,87,88]. Signal processing methods diagnose faults by analyzing various signals generated during the operation of robots, such as vibration, current, sound, and temperature [66,89,90,91,92]. Common signal processing techniques include Fourier transform, wavelet transform, variational mode decomposition, local mean decomposition, etc. [93,94,95,96,97]. Jiang et al. [95] investigated a method that combines empirical wavelet transform with chaotic oscillators. By using the empirical wavelet transform, the composite fault signal is decomposed into different empirical modes. A fault isolator is then established using an oscillator that includes all individual fault frequencies. Finally, all fault types are identified by observing the irregular motion generated from the mapping of the isolator’s output. Wan et al. [96] combined fast spectral kurtosis with variational mode decomposition to process compound fault signals containing weak single components. Zhao et al. [97] investigated a fault signal processing method based on local mean decomposition, considering the local non-stationary characteristics of vibration signals. By defining a non-stationary coefficient to characterize the local non-stationary features of vibration signals, they proposed a composite envelope construction method. This method involves using monotonic segment cubic Hermite interpolation for the non-stationary parts and cubic spline interpolation for the smooth parts. The local mean decomposition algorithm designed based on this envelope method is applied to fault diagnosis.

For the traditional methods, although these methods have shown some effectiveness in the early fault diagnosis of manipulator robots, with the increase in the complexity of industrial scenes, the accuracy and stability of the traditional methods are limited.

### 3.2. Based on Modern Methods

**Figure 5:**
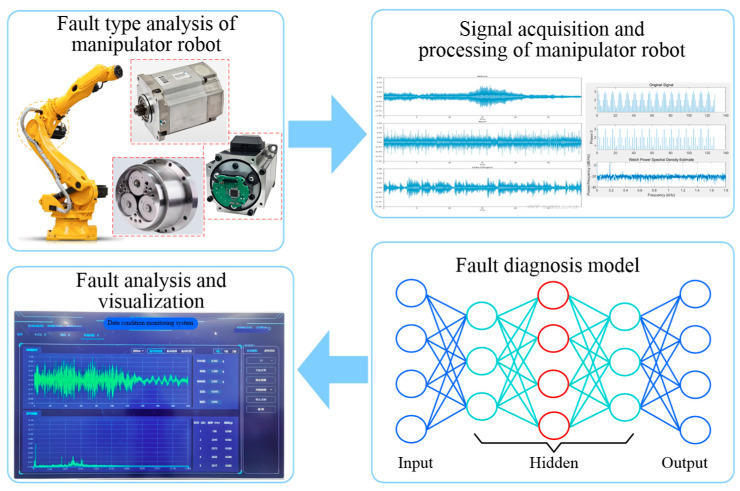
Manipulator robot fault diagnosis based on modern methods.

In recent years, the rapid development of emerging technologies such as artificial intelligence, big data, cloud computing, Internet+, and information fusion has stimulated breakthroughs in manipulator robot fault diagnosis technology [98,99]. By constructing data-friendly signal processing methods to extract features that differ from normal information, fault-related information can be obtained [100]. Then, a fault diagnosis model can be constructed using deep learning or machine learning methods and displayed through a visual interface, as shown in Figure 5.

#### 3.2.1. Method Based on Artificial Intelligence

**Figure 6:**
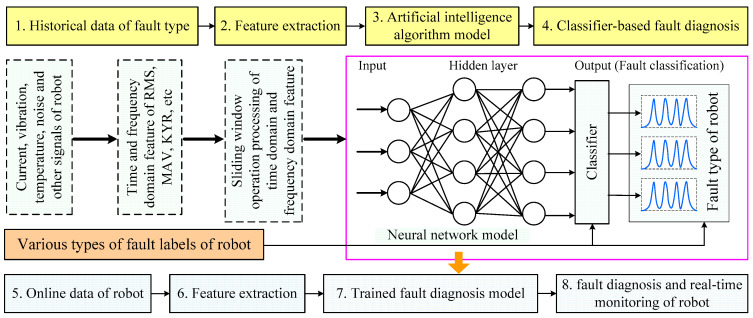
Fault diagnosis process based on artificial intelligence method.

The resurgence of neural networks and the rise of machine learning have led to breakthroughs in fault diagnosis research [101,102]. Manipulator robot fault diagnosis methods based on neural networks have also become increasingly popular. These methods use historical data that has been collected and subjected to feature selection to train various types of learning algorithms, thereby identifying compound fault patterns. Finally, online data is used as input to the model for fault diagnosis, and the process is illustrated in Figure 6. For example, Bai et al. [103] proposed a method for screening fault data and a fault rate diagnosis framework for manipulator robots. They established a fault rate model for manipulator robots with different subsystems, then used a surrogate model to fit the bathtub curve of the original robot to obtain the early failure time points. The distribution parameters of the original manipulator robot were solved using the maximum likelihood function to quantify the influencing factors of the manipulator robot. Finally, the interval hierarchical analysis method was used to refine the cognitive uncertainty and obtain the correction factor for the fault rate. Although methods based on traditional neural networks offer certain advantages for fault prediction in manipulator robots, limited sample sizes can result in poor generalization performance of these networks.

Artificial intelligence methods, such as CNN [104] and GNN [105], have brought significant changes to the field of signal processing and are receiving considerable attention in the field of fault diagnosis. These methods can increasingly accurately fit the nonlinear relationships between samples and label values through continuous sample training. Moreover, by continuously learning from historical and new fault data, it can construct and optimize fault diagnosis models. These methods require minimal human intervention and have advantages in handling complex, nonlinear problems. Lu et al. [11] proposed a fault diagnosis method for manipulator robot reducers using dual module attention CNNs. This method captures different features related to faults by establishing two parallel CNNs with different focuses. Finally, the fault features are fused to obtain the fault diagnosis result (normal or abnormal). Jiao et al. [106] proposed a fault diagnosis method based on deep belief network (DBN) joint information fusion technology to overcome the problems of low accuracy, low efficiency, poor stability, and poor real-time performance of traditional manipulator robot fault diagnosis models in multi-fault state diagnosis. This method, combining DBN and wavelet energy entropy, is used to study the fault diagnosis of manipulator robots. It uses wavelet transform to denoise, decompose, and reconstruct vibration signals of manipulator robot joint bearings, and establishes normalized eigenvectors for reconstructing energy entropy and using them as inputs to DBN. The use of improved D-S evidence theory has solved the problem of eliminating high-conflict evidence and improved the recognition accuracy of fault models. Yang et al. [8] proposed a fault diagnosis method for manipulator robots in dynamic working states. This study utilizes a domain generalization adversarial long short-term memory residual life prediction modeling method to reduce differences between robots. The two-stage health assessment method of principal component analysis prediction error square and p-chart is used to reduce the interference of outliers in the normal operation of robots. The method uses feature smoothing, feature normalization, and feature selection to reduce changes in the working area, thereby effectively reducing errors.

The working state of manipulator robots is affected by external factors, which can lead to a decrease in the accuracy and stability of fault diagnosis models. In addition, the differences between different robots require large-scale adjustments to the fault diagnosis model during deployment. Adaptive learning or transfer learning allows models to dynamically adjust their parameters and structure based on new data, and can quickly improve diagnostic accuracy using previous training experience [107,108]. To address the issue of variable working conditions in manipulator robots, Xia et al. [48] proposed a novel Deep Perceptual Adversarial Domain Adaptation method for fault diagnosis of manipulator robot joint bearings under different operating conditions. This approach employs a novel perception loss based on information entropy to enforce identical distributions between the source and target domains, thereby enhancing the stability of adversarial training. To address the limitations of traditional fault detection methods that require extensive labeled data and exhibit poor generalization capabilities, Kumar et al. [109] proposed a transfer learning-based fault detection method for servo motor bearings, which significantly improves detection efficiency and accuracy in new bearing fault diagnosis tasks by transferring knowledge from pre-trained CNN models. Transfer learning or adaptive learning methods adapt models through prior training experience without requiring full retraining, enabling more efficient deployment across diverse manipulator robot fault diagnosis platforms.

#### 3.2.2. Method Based on Digital Twin Technology

**Figure 7:**
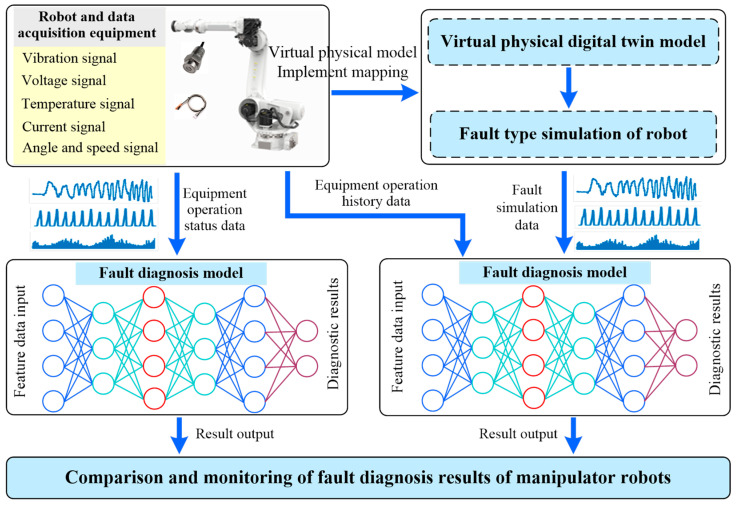
Fault diagnosis architecture for manipulator robots based on digital twins.

Recently, a new solution has been provided for the fault diagnosis of manipulator robots: digital twin technology. As an emerging cutting-edge technology, digital twin technology utilizes tools such as CAD software(SolidWorks 2022, Dassault Systèmes, France) [110] to create high-fidelity digital models in virtual space that correspond to physical objects and combines network communication technology to achieve real-time mapping and interaction between the physical world and the digital world [111,112,113]. Digital twin technology provides new perspectives and methods for the operation and maintenance of manipulator robots, which helps improve the accuracy and real-time performance of fault diagnosis for manipulator robots. The fault diagnosis scheme for manipulator robots based on digital twin technology is shown in Figure 7.

Song et al. [114] proposed a manipulator robot joint fault diagnosis system assisted by a twin subsystem. Data generated from the robot dynamics model is utilized as the virtual entity data for the digital twin model. Through the proposed CycleGAN-based digital twin model, a minimal amount of empirical data is employed to map the virtual entity data onto the physical entity. The validity of the simulation data generated by the digital twin model is corroborated using a CNN-ResNet classifier. Ademujimi et al. [115] discussed the application of digital twin technology in fault diagnosis for manipulator robots. The study constructed a co-simulation model that includes network sensors, high-fidelity simulation models, and discrete event simulation models. A structural intervention algorithm is designed to detect directed edges in Bayesian networks and to distinguish between parent nodes and ancestor nodes. The effectiveness of the proposed method was validated through experiments.

#### 3.2.3. Method Based on Multi-Information Fusion

**Figure 8:**
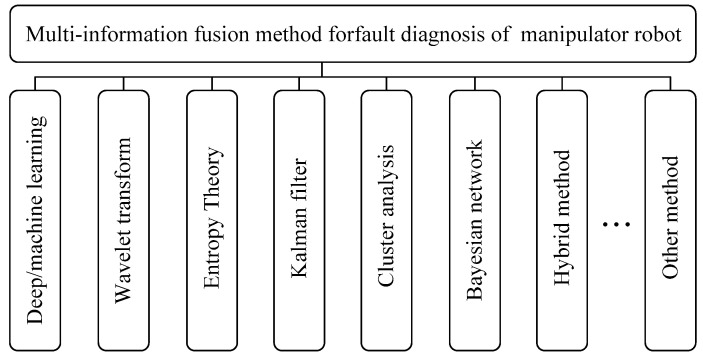
Multi-information fusion methods commonly used in fault diagnosis of manipulator robots.

Multi-information fusion is similar to imitating the decision-making process of the brain, perceiving the state information of objective objects through multiple senses, and then conducting joint decision-making and comprehensive analysis through information integration, thereby continuously increasing the cognitive level of objective objects and achieving the goal of recognition. In the diagnostic research of manipulator robots, due to the complexity of the equipment and the harshness of the operating environment, information from single-source sensor devices often fails to extensively extract the global features of faults [116]. This inevitably leads to a reduction in the accuracy of fault diagnosis, and may even result in missed diagnoses and incorrect diagnoses [117]. Multi-information fusion technology provides a new approach to solving the uncertainty problem in mechanical fault diagnosis. In the field of fault diagnosis, multi-information fusion technology comprehensively collects equipment fault status information through multiple sensors and mines the coupling and complementary information between multi-source sensor data. By using multi-feature fusion analysis and joint decision-making as means, it improves the reliability and accuracy of fault diagnosis, overcoming the disadvantages of limited fault information and uncertainty inherent in single sensors [118]. The multi-information fusion method has been widely used in the fault diagnosis of manipulator robots [119,120,121,122,123], as shown in Figure 8.

Gong et al. [117] proposed a hierarchical visual transformer and wavelet time-frequency multi-source information fusion intelligent fault diagnosis method for mechanical components. This method introduces a hierarchical visual transformer to improve feature mapping representation, enriching fault features. It fuses two-dimensional time-frequency diagrams of multi-source signals into the proposed hierarchical visual transformer for a more comprehensive representation of fault features, thereby enhancing the effectiveness of fault diagnosis. Suawa et al. [53] fused vibration and sound data collected by sensors installed on the motor, and analyzed the data through a deep CNN to diagnose faults, thereby addressing the issue of predictive maintenance for motors. Hoang et al. [100] proposed a fault diagnosis method that utilizes deep learning and information fusion techniques. This method primarily collects raw signals from multiple phases of the motor current as direct inputs and extracts features from the current signals of each phase. Then, a CNN is employed to classify each feature set. A novel decision-level information fusion technique is introduced to integrate information from all CNNs, transforming the decision-level information fusion problem into a pattern classification task to obtain accurate fault diagnosis results.

#### 3.2.4. Other Methods for Manipulator Robot Fault Diagnosis

There are also some other combination methods used for fault diagnosis in manipulator robots, which overcome the shortcomings of single methods by combining multiple different forms of methods [124,125,126]. Composite structure is a proven method. For instance, Yin et al. [125] addressed the issue of integrating prior knowledge with learning networks by proposing a fault diagnosis transfer network driven by both knowledge and data. They extracted prior knowledge from fault signals through envelope analysis and passband selection and used convolutional kernels and designed filters to integrate this prior knowledge. The network employs a deep adaptive network embedding with weight sharing to embed and transfer prior knowledge. Bilal et al. [126] proposed an Internet of Robotic Things (IoRT) architecture based on transfer learning technology for fault detection of industrial manipulators. This method employs a hybrid 1-D multichannel CNN (MCNN) that combines matrix kernels and RNN to achieve high-precision detection of robot joint states. Furthermore, the study introduced a variable working condition joint fault diagnosis algorithm based on transfer learning with matrix kernels and recurrent neural networks, overcoming the challenges of fault diagnosis for manipulator robots under complex working conditions.

Cross-mode or cross-domain fusion, as a novel method, is applied in the research of fault diagnosis for manipulator robots [127,128,129,130,131]. Ref. [128] proposes a semantic-aware cross-domain industrial process fault diagnosis network based on knowledge mining. The network designs a non-shared attention mechanism to obtain discriminative features for each operating condition. It constructs a fault relationship knowledge graph through a cross-correlation knowledge mining subnetwork to explicitly constrain the local consistency between the source domain, target domain, and cross-domain. A semantic knowledge transfer subnetwork is utilized to impose semantic constraints during the knowledge transfer process. Ultimately, the subnetworks are jointly trained for cross-domain industrial process fault diagnosis. Xu et al. [130] proposed a cross-modal fusion CNN for mechanical fault diagnosis. This network employs two parallel modality feature networks to learn specific modality features of independent signals and a cross-modal knowledge-sharing network to learn shared features from multi-source data. The fault diagnosis performance of the cross-modal fusion convolutional neural network is then enhanced through the online soft-label training strategy. Lee et al. [131] addressed the issue of low prediction confidence in fault diagnosis models in the industrial domain by proposing an interpretable fault diagnosis method that incorporates domain knowledge. This method utilizes the interpretability of generated fuzzy energy pattern image data to infer the causal relationships of faults.

Modern methods have excellent performance in fault diagnosis of manipulator robots, but their application is still limited by factors such as data and computing resources. These limitations will be discussed in the Section 4.

## 4. Discussion

This paper summarizes the fault types and causes of manipulator robots, as well as the research status of fault diagnosis methods. Table 2 and Table 3 respectively list the typical papers of traditional and modern methods for fault diagnosis of manipulator robots.

### 4.1. Summary and Analysis

The research on fault diagnosis of manipulator robots is a challenging research topic [132]. To achieve the safe and effective operation of manipulator robots, it is urgent to improve the accuracy of manipulator robot fault diagnosis algorithm recognition and explore innovative fault diagnosis technologies. Although some traditional statistical and mechanism knowledge-driven methods can achieve fault diagnosis of manipulator robots, the target objects are mostly single faults. Due to the complex working environment, manipulator robot failures are mostly compound, and the accuracy of detecting compound failures through traditional methods is insufficient. For example, it is difficult to acquire and update the knowledge of expert systems, and the statistical model relies heavily on the assumption of data distribution, which limits its application in complex industrial scenes. In recent years, with the rapid development of artificial intelligence and IoT technology, the use of advanced methods such as deep learning, multi-information fusion, and digital twins for manipulator robot fault diagnosis have attracted attention. Although multi-information fusion methods can significantly improve the accuracy of fault diagnosis in manipulator robots, the time alignment of data collected by multiple sensors, the uniformity of data format, and the computational time cost of deep learning algorithms can affect the overall performance of fault diagnosis. Moreover, the deep learning method has poor interpretability, high requirements for the amount of data, and high model training costs. Although digital twins can realize the fusion of the virtual and the real, they still face the challenges of data synchronization and model construction in practical application. Therefore, further research is needed to enhance the speed and stability of new technologies used for fault diagnosis.

Finally, we summarize the fault diagnosis methods for manipulator robots into two aspects: Firstly, the advantages and disadvantages of using traditional methods such as statistics, mechanism-knowledge-driven, and signal processing for fault diagnosis are analyzed. Secondly, modern methods represented by artificial intelligence and digital twins were analyzed.

**(1) Traditional methods for fault diagnosis:** Traditional methods for fault diagnosis of manipulator robots have some advantages, but there are still limitations in practical applications.

**Advantages:** These methods are easy to implement, have strong anti-interference ability, and have good stability in diagnosing a single fault.

**Limitations:** Insufficient accuracy in compound fault diagnosis of manipulator robot systems.

**(2) Modern methods for fault diagnosis:** Similarly, modern methods for manipulator robot fault diagnosis also have advantages and limitations.

**Advantages:** High accuracy in identifying composite faults, and the equipment can be visualized for remote monitoring.

**Limitations:** The data formats and timing from multi-sensor acquisition are difficult to unify, and deep learning training requires a large number of samples with high training time costs. Signals are easily affected by the acquisition environment, noise, and other factors, leading to insufficient information stability and affecting the accuracy of fault diagnosis. Moreover, the use of digital twin technology for fault diagnosis imposes higher requirements on communication technology, data storage, and computation, and the real-time requirements for visualization and monitoring demand that the hardware system has powerful computational performance.

Traditional and modern methods have advantages and limitations in the fault diagnosis of manipulator robots. For example, in some specific operating conditions, especially for manipulator robot failure modes with periodic signals or known characteristics or single fault types, traditional methods may perform well. However, for the fault diagnosis of manipulator robots in complex nonlinear signals or high-noise environments, traditional methods cannot effectively distinguish fault types and rely on manually designed features. Modern methods have high accuracy in diagnosing complex signals and multiple types of faults and can better handle nonlinear data. However, modern methods such as deep learning are highly dependent on data and have higher computational resource requirements compared to traditional methods, limiting real-time performance. It is worth noting that in modern manufacturing, due to the complexity of tasks and the increasing demand, a single method may not be able to meet actual needs, and it is necessary to choose the appropriate method or a combination of two methods based on reality. The comparison between traditional and modern methods in terms of accuracy, stability, and rapidity is shown in Table 4.

Some solutions have the potential to address the aforementioned limitations.

**Semi-supervised learning:** By integrating a semi-supervised learning mechanism, the model training process effectively mitigates the challenge of limited training data by leveraging scarce labeled samples and abundant unlabeled data.

**Multi-sensor data fusion optimization:** Improve the efficiency and accuracy of data fusion by improving data preprocessing methods (such as wavelet transform, and PCA dimensionality reduction) and introducing more efficient data fusion algorithms (such as time series analysis based on informer).

**Model optimization and improvement:** Through transfer learning and meta-learning, to improve the adaptability of the model under different working conditions.

**Hardware support and algorithm optimization:** Combine edge computing devices (such as high-performance PLC) and optimized algorithm architecture (such as lightweight neural network), reduce computing costs, and improve real-time performance

In summary, both traditional and modern methods have their advantages and disadvantages in practical applications. Next, we will explore the future development direction of manipulator robot fault diagnosis.

### 4.2. Future Expectations

The fault diagnosis technology of manipulator robots plays an important role in intelligent manufacturing and industrial production. To improve the effectiveness and applicability of its practical application, the development of fault diagnosis for manipulator robots should meet the following requirements:

**Intelligentization:** Use advanced technologies such as deep learning and cloud computing, combined with high-performance edge computing equipment, to improve the computational power and speed of the model, and improve the intelligent level of fault diagnosis.

**Integration:** Integrate fault diagnosis technology more closely with the control system and sensor system of the manipulator robot, and combine the mechanism model and artificial intelligence method of the robot to realize online monitoring and real-time early warning.

**Servitization:** Develop services based on the cloud platform, combine manipulator robot and IoT, and use advanced communication technology to build a high fidelity, real-time, and accurate manipulator robot human networking and visualization system (manipulator robot digital twin system) to provide users with remote fault diagnosis and prediction services.

**Quantitative evaluation:** According to the application scenarios, application objects, and application environments of different manipulator robots, the corresponding quantitative fault evaluation system should be formulated and improved to promote the wide application of technology.

The first requirement is a prerequisite for achieving accurate fault diagnosis of manipulator robots. By integrating some advanced artificial intelligence algorithms and new IoT technologies, it is possible to effectively combine the operational status data of manipulator robots and extract effective fault feature information. This makes it easier for fault diagnosis models to understand the robot’s data, thereby improving the accuracy of fault diagnosis results. The second requirement, building upon the first, involves integrating intelligent diagnostic methods with the inherent systems of manipulator robots. By complementing each other through the robot’s mechanism and artificial intelligence methods, it achieves online monitoring of the robot’s operational status and fault prediction. The third requirement is a further enhancement, which aims to construct a high-fidelity digital twin for the manipulator robot fault diagnosis system through advanced cloud platforms, industrial internet, and modern communication technologies. This provides technicians with remote visualization of equipment operational status, enabling remote fault diagnosis and predictive services for manipulator robot systems. Furthermore, manipulator robots are applied in a wide range of scenarios with complex application environments, making it difficult to assess the causes and severity of faults under different operating conditions. At present, such assessments are mostly made based on extensive on-site experience or whether the robot can operate normally. Quantitative assessment of manipulator robot fault diagnosis is crucial for establishing and refining fault evaluation systems that correspond to different scenarios and environments. This is of significant importance for utilization in industrial production.

## 5. Conclusions

Research on fault diagnosis of manipulator robots has attracted increasing attention from both scholars and the industrial community. Establishing a robust fault diagnosis technology system for robots can accurately pinpoint potential faults before they manifest during the production process. This facilitates equipment maintenance and enables early warning and proactive management of potential fault risks, thereby reducing labor costs and enhancing production efficiency. This paper reviews the current research status and major methods of fault diagnosis for manipulator robots. Through comparative analysis, it can be observed that new methods based on artificial intelligence, digital twins, and multi-information fusion have shown great potential and advantages in fault diagnosis. It is concluded that the future development of manipulator robot fault diagnosis may benefit from the following considerations.

(1) Efficient, accurate, and stable fault diagnosis is the foundation for the practical production application of manipulator robots.
(2) The integration of multi-information fusion and artificial intelligence algorithms can compensate for the shortcomings of single signals that fail to effectively reflect fault characteristics of manipulator robots, as well as the challenges posed by traditional algorithms in handling composite faults. However, improvements are still needed in data fusion strategies and the computational time cost of models.
(3) Digital twin technology can achieve remote monitoring of the operational status, fault diagnosis, and predictive maintenance of manipulator robot systems.
(4) Establishing and refining a quantitative assessment system for fault diagnosis of manipulator robots under different operating conditions and environments can enhance production efficiency and reduce production costs.

There is still a long way to go to achieve the aforementioned objectives. However, these methods will become more mature and widespread with the enhancement of computational capabilities and the advancement of big data technologies. Furthermore, although this review systematically discusses the types of faults and fault diagnosis methods for manipulator robots, there are still certain limitations. For fault diagnosis of manipulator robots, the cost of multi-information acquisition and the performance requirements of computational devices for data cleaning and diagnostic models have not been mentioned. These limitations all involve factors that affect the research on fault diagnosis of manipulator robots.

## Figures and Tables

**Table 2:** Summary of typical papers on traditional methods.

Form of Method	Sensors	References	Purpose of Research	Key Features of the Research
Expert experience	Robot data and vision sensor	[76]	Avoiding misdiagnosis and missed diagnosis of robot fault	Estimate the motion state of the robot using a Kalman filter and calculate the probability of the corresponding fault mode for the motion state; selecting appropriate knowledge from expert experience systems to diagnostic results
	Vibration measurement sensor	[77]	Strategy and full load working environment, bearing fault diagnosis	Propose a new method of random fuzzy evidence acquisition and intuitionistic fuzzy set fusion by constructing a fuzzy expert system and matching the samples to be tested
Statistic model	Vibration measurement sensor	[78]	A lot of redundant information in the vibration signal, cannot be directly used for fault diagnosis without processing	Principal component analysis, locality preserving projection and isometric feature mapping extract three-dimensional features of vibration signals and form nine-dimensional features, and train naive Bayesian model to identify fault types
Mechanism knowledge-driven	Motor encoder feedback	[80]	Uncertain and nonlinear effects of manipulator robot operating environment fault diagnosis and fault-tolerant control	The neural network adaptive high-order variable structure fault diagnosis observer based on machine learning improves the robustness of fault signal estimation; adaptive modern fuzzy backstepping variable structure controller to reduce the impact of faults on the robot
	Motor encoder feedback	[81]	Fault diagnosis of uncertain and nonlinear effects of manipulator robot operating environment	Using the observer with unknown input, combined with the estimated virtual mode, the residual is generated to detect and diagnose the fault information of the robot
	Image sensor	[84]	Detecting and isolating potential faults in manipulator robots	Kalman filter estimates the system state and calculates the residual between the actual image feature output and the Kalman filter estimation, and determines the fault based on the residual threshold
	Angle sensor and camera	[85]	Improve the safety and robustness of the manipulator when using only position signals	Fault diagnosis and fault-tolerant control methods for adaptive extended Kalman filter and sliding mode, detecting faults and fault-tolerant control of manipulator
Signal Processing	Vibration sensor (accelerometers)	[95]	Different faults occur simultaneously; compound fault decoupling detection is difficult	The empirical wavelet transform uses an adaptive wavelet basis to extract the intrinsic mode of the signal and decomposes the compound fault into single fault with different empirical modes. Each fault is merged into a continuous oscillator, and the fault type is identified by observing the irregular motion generated by the output of the established isolator.
	vibration sensor (accelerometers)	[96]	The faults of bearings are located in different resonance bands and the mutual interference and noise influence between different fault components	The original composite fault signal is decomposed and preprocessed by the variational mode decomposition method, which is decomposed into multiple variational eigenmode function components, and the eigenmode function components are calculated by fast spectral kurtosis
	ICP acceleration sensor	[97]	Vibration signal has nonlinear, non-stationary, multi-component coupling characteristics, and traditional methods cannot effectively extract the characteristics of the vibration signal	Compound interpolation CIE LMD method: nonstationary coefficient is defined to represent the local nonstationarity of vibration signal, monotone piecewise cubic Hermite interpolation is used for the nonstationary part, and cubic spline rubbing is used for the smooth part

**Table 3:** Summary of typical papers on modern methods.

**Form of Method**	**Sensors**	**References**	**Purpose of Research**	**Key Features of the Research**
Artificial intelligence	Motor encoder feedback	[11]	Current methods pay little attention to the correlation and internal differences of test data	Two parallel convolutional neural networks with different attention mechanisms are established to obtain different fault-related features
	vibration measurement sensor	[106]	Low accuracy and efficiency, poor stability and real-time performance of manipulator robot multi-fault state diagnosis	The deep confidence network is combined with wavelet energy entropy; wavelet transform is used to denoise, decompose, and reconstruct the vibration signal, and the normalized eigenvector of reconstructed energy entropy is used as the input of deep confidence network
	Robot controller	[8]	Difficulty in fault diagnosis under dynamic working state	Domain generalization-residual life prediction against long short-term memory, principal component analysis squared prediction error, and p-chart to reduce abnormal interference
Digital twins	Acceleration sensor, Temperature sensor	[114]	The actual joint fault data of the robot is insufficient, which is difficult to obtain in real-time	Based on the digital twin model of CycleGAN, the virtual model is mapped to the physical entity using a small amount of measured data, and the joint state data is obtained in real time
	Vibration measurement sensor; Temperature sensor	[115]	Lack of data and computing power makes it difficult to develop a universal high-fidelity engineering digital twin model for complex systems	Engineering digital twin system: use network physical system to form network sensor and build equipment simulation model; build discrete event simulation model factory; engineering digital twin method injects faults into the virtual system for equipment and plant level fault diagnosis
Multi-information fusion	Accelerometer; Current sensor	[117]	The existing methods cannot effectively capture the time information and global characteristics of the device; Single source fault diagnosis method makes it difficult to accurately extract fault features	Intelligent fault diagnosis method based on multi-source information fusion of hierarchical visual transformer and wavelet time-frequency; the multi-source signals are converted into two-dimensional time-frequency maps and fused into a hierarchical visual transformer
	Accelerometer, Microphone	[53]	The multi-sensor data combination can observe the phenomenon of more abundant system degradation, which is helpful for equipment analysis and decision-making	The influence of data level fusion on the accuracy of different detection tools is studied, and the best combination example of sensor and deep learning fusion algorithm is studied to achieve accurate predictive maintenance; build DCNN, LSTM, and CNN-LSTM models
	Motor polyphase current signal	[100]	Some systems cannot install external sensors; the bearing installed outside the motor cannot effectively measure the current	Taking the multi-phase original signal of motor current as input, the characteristics of each phase current are extracted, and the features are classified by CNN. The decision-making level information fusion technology converts CNN information into simple pattern classification problem
Other method	Accelerometer	[125]	The effective value of prior knowledge and learning network is not mined in the fusion, which reduces the performance of fault diagnosis	Extracts the prior knowledge of the fault signal through envelope analysis and passband selection, fuses the prior knowledge with the convolution kernel and the designed filter, embeds and transmits the prior knowledge and cross-domain knowledge using the weight sharing depth adaptive network, and uses the maximum average deviation of multiple cores for regional adaptation
	Vibration sensor	[126]	The method can self-maintain, diagnose and repair, and carry out reliable and robust online monitoring for robots	The method based on transitive learning makes use of the inherent relationship between the adaptability of transitive learning and different failure modes; hybrid one-dimensional MCNN based on matrix kernel and RNN is used to realize the high-precision detection of robot state; time stamp mapping to overcome time inconsistency of multi-sensor
	Machine monitoring process signal	[128]	Difficulty in obtaining fault samples under changing operating conditions and ineffective application or performance degradation in actual industry; Not utilizing the internal and relevant knowledge under different working conditions	A non-sharing mechanism is designed to obtain the discriminant characteristics of each working condition; the cross-correlation knowledge mining subnet is used to construct the fault relationship knowledge graph to explicitly constrain the local consistency between the source domain, target domain, and cross-domain; semantic knowledge transfer subnet makes output consistent and distinguishable
	Vibration sensors, Sound sensors	[130]	The intrinsic correlation between the distribution gap of CNN and multi-frequency mechanical signals in the learning process is not considered	Two parallel modal-specific networks and a cross-modal knowledge-sharing network are used to explore the independent and shared features of multi-modal mechanical signals; cross-modal fusion is introduced to fuse and transfer the cross-modal features to the next layer
	Accelerometer, Laser sensor, Current sensor, Voltage probe	[131]	Low confidence in robot fault model prediction in the industrial field	Inferring the causal relationship of faults by using the physical interpretability of generating fuzzy energy pattern image data

**Table 4:** Comparison between traditional and modern methods.

**Characteristic**	**Traditional Methods**	**Modern Methods**
Accuracy	Excellent performance in single failure mode or periodic signal, relying on manual feature design;	Excellent performance in complex signals and multiple types of fault modes, but strong data dependence
Stability	Simple method deployment; known working conditions perform well	Strong adaptability to complex working conditions, but requires a large amount of data support
Rapidity	Fast calculation speed, suitable for real-time fault diagnosis	High demand for computing resources, real-time fault diagnosis relies on high-performance hardware devices

## Data Availability

Not applicable.

## Abbreviations

RV	Rotary Vector
CNN	Convolutional neural network
GCN	Graph convolutional network
LSTM	Long short-term memory
DBN	Deep belief network
MCNN	Multichannel convolutional neural network
IoRT	Internet of robotic things
PCA	Principle component analysis
PLC	Programmable logic controller
CAD	Computer aided technology
